# Predictive Value of Umbilical Artery Half Peak Systolic Velocity Deceleration Time for Adverse Perinatal Outcomes in Gestational Diabetes Mellitus

**DOI:** 10.3390/jcm14197016

**Published:** 2025-10-03

**Authors:** Ruken Dayanan, Dilara Duygulu Bulan, Merve Ayas Ozkan, Gulsan Karabay, Zeynep Seyhanli, Ali Turhan Caglar

**Affiliations:** Department of Obstetrics and Gynecology, Division of Perinatology, Ankara Etlik City Hospital, 06170 Ankara, Turkey; duyguludilara4@gmail.com (D.D.B.); merveayasozkan@gmail.com (M.A.O.); drgulsankarabay@gmail.com (G.K.); drzeynepseyhanli@gmail.com (Z.S.); turhan_caglar@yahoo.com (A.T.C.)

**Keywords:** gestational diabetes mellitus, umbilical artery, half peak systolic velocity deceleration time, Doppler ultrasound, adverse perinatal outcome

## Abstract

**Objective:** To evaluate the predictive value of umbilical artery half peak systolic velocity deceleration time (UA hPSV-DT) for composite adverse perinatal outcomes (CAPO) in pregnancies complicated by gestational diabetes mellitus (GDM). **Methods:** In this prospective observational study, 120 singleton pregnancies in the third trimester were enrolled: 30 insulin-regulated GDM (IRGDM), 30 diet-regulated GDM (DRGDM), and 60 healthy controls. UA hPSV-DT and standard Doppler indices were measured using a standardized protocol by a single perinatologist. An abnormal UA hPSV-DT was defined as <5th percentile for gestational age. Maternal metabolic parameters, fetal biometry, and neonatal outcomes were recorded. The primary outcome was CAPO, defined as the presence of one or more adverse perinatal events. **Results:** Median UA hPSV-DT values were significantly lower in IRGDM (171 ms) and DRGDM (184 ms) compared with controls (227 ms) (*p* = 0.006). Abnormal UA hPSV-DT occurred in 43.3% of GDM cases and was associated with higher estimated fetal weight and abdominal circumference percentiles, increased amniotic fluid, elevated OGTT values, higher HbA1c, and more frequent insulin therapy (*p* < 0.01 for all). In GDM pregnancies, CAPO occurred in 73.1% of the abnormal UA hPSV-DT group versus 11.8% of the normal group (*p* < 0.001). ROC analysis identified a cut-off of < 181 ms for predicting CAPO (AUC 0.741, 70.3% sensitivity, 66.7% specificity). **Conclusions:** UA hPSV-DT is a novel, reproducible Doppler parameter that independently predicts adverse perinatal outcomes in GDM pregnancies, even when conventional UA Doppler indices are normal. Incorporating UA hPSV-DT into routine surveillance may improve risk stratification and guide management to optimize perinatal outcomes.

## 1. Introduction

Gestational diabetes mellitus (GDM) is a common pregnancy complication, affecting roughly 6–15% of pregnancies worldwide [1]. It is characterized by glucose intolerance leading to maternal hyperglycemia, which exposes the fetus to excess glucose and provokes fetal hyperinsulinemia and accelerated growth (macrosomia) [2,3,4]. Beyond these metabolic effects, GDM induces important cardiovascular and hemodynamic changes: chronic hyperglycemia and insulin resistance can impair maternal endothelial function and placental angiogenesis, thereby altering uteroplacental blood flow [5,6,7]. Doppler studies indicate that diabetic pregnancies tend to have higher indices of placental vascular resistance (e.g., elevated umbilical artery resistance index and uterine artery pulsatility index) compared to normoglycemic pregnancies [8]. Such hemodynamic disturbances contribute to the increased risk of adverse perinatal outcomes in GDM—including preeclampsia, fetal distress, and even stillbirth—which are thought to stem in part from placental dysfunction.

One emerging Doppler parameter to assess fetoplacental hemodynamics is the umbilical artery half-peak systolic velocity deceleration time (UA hPSV-DT). This index quantifies the time (in milliseconds) for the umbilical artery blood flow velocity to decelerate from its systolic peak to half of that peak. Notably, the measurement is largely independent of fetal heart rate and has been shown to be highly reproducible, with low intra- and inter-observer variability [9]. Physiologically, UA hPSV-DT reflects the runoff of blood through the placenta: a shorter deceleration time indicates a rapid fall in velocity due to high downstream resistance or reduced placental compliance, whereas a longer deceleration time suggests lower resistance and more sustained diastolic flow. Indeed, UA hPSV-DT is inversely correlated with the conventional umbilical artery pulsatility index and has been validated as an index of placental vascular resistance [10]. In clinical research, this novel Doppler metric has already shown promise in characterizing placental circulation; for example, in fetal growth restriction, abnormally low UA hPSV-DT values were closely associated with poor perinatal outcomes.

Given the potential impact of GDM on placental vascular structure and function, there is a strong physiological basis for exploring UA hPSV-DT as an indicator of fetoplacental health in this context. Chronic maternal hyperglycemia may impair villous vascular development and increase downstream placental resistance, changes that could be sensitively detected by a shortened UA hPSV-DT. Detecting such alterations before conventional Doppler indices become abnormal could allow earlier identification of pregnancies at risk for compromised fetal well-being. We hypothesize that reduced UA hPSV-DT values are associated with an increased risk of adverse perinatal outcomes in pregnancies complicated by GDM. The aim of this study is therefore to evaluate the predictive value of UA hPSV-DT for composite adverse perinatal outcomes in women with GDM and to determine whether this novel Doppler parameter can enhance risk stratification beyond standard clinical and sonographic assessments.

## 2. Materials and Methods

This prospective observational study was conducted at the Department of Perinatology, Ankara Etlik City Hospital, between February 2025 and August 2025. A total of 120 singleton pregnant women in their third trimester were included. Sample size was determined a priori using G*Power (version 3.1.9.7; Heinrich-Heine-Universität Düsseldorf, Düsseldorf, Germany), based on an assumed effect size that would provide 80% power at a two-sided α = 0.05. The study population was divided into three groups: 30 patients with insulin-regulated gestational diabetes mellitus (IRGDM), 30 patients with diet-regulated gestational diabetes mellitus (DRGDM), and 60 healthy pregnant women as the control group. Gestational age-matched controls were recruited from routine antenatal visits to minimize confounding.

The diagnosis of GDM was made according to the International Association of the Diabetes and Pregnancy Study Groups (IADPSG) criteria, using a 75 g oral glucose tolerance test (OGTT) performed between 24 and 28 weeks of gestation [11]. Venous plasma glucose was measured at fasting, 1 h, and 2 h after the glucose load, and GDM was diagnosed if at least one of these values met or exceeded the established thresholds: fasting ≥ 92 mg/dL, 1 h ≥ 180 mg/dL, or 2 h ≥ 153 mg/dL. Exclusion criteria for all groups included pregestational diabetes mellitus, chronic hypertension, autoimmune or inflammatory disorders, renal or hepatic disease, multiple pregnancy, fetal anomalies, known chromosomal/genetic abnormalities, smoking, and alcohol use. Women with incomplete clinical or ultrasound data were also excluded.

**Figure 1:**
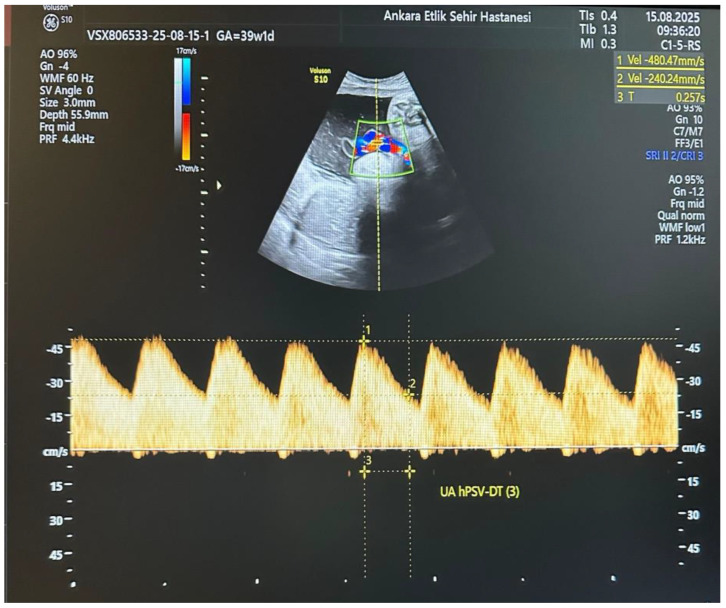
Umbilical artery half peak systolic velocity deceleration time (UA hPSV-DT): a novel Doppler parameter for prediction of adverse perinatal outcomes in pregnancies complicated by gestational diabetes mellitus. Measurement of the umbilical artery half-peak systolic velocity deceleration time (UA hPSV-DT) from a free-floating loop of the umbilical cord at 39 + 1 weeks of gestation. The Doppler insonation angle was kept below 30° during fetal quiescence without breathing movements. The calipers were placed at the peak systolic velocity and at half of this peak, and the time interval between these points was recorded in milliseconds according to the method of Bustos et al.

All participants underwent detailed ultrasound and Doppler examinations using a Voluson E10 system (GE Healthcare, Zipf, Austria) with a 2–6 MHz convex transducer. All measurements were performed by a single experienced perinatologist using a standardized protocol. The operator was blinded to maternal metabolic parameters (HbA1c, OGTT results, treatment type) at the time of Doppler assessment. Umbilical artery (UA) Doppler waveforms were obtained from a free-floating cord loop during fetal quiescence and in the absence of fetal breathing movements, ensuring an insonation angle < 30°. The UA hPSV-DT measurement was performed as described by Bustos JC et al. [10]. On the selected UA waveform, the first caliper was placed at the peak systolic velocity, and the second caliper at the midpoint between the baseline and the peak velocity. A straight line was then drawn from the second caliper to intersect the spectral envelope, and the time interval between these two points was measured in milliseconds (Figure 1). This process was repeated for three consecutive uniform waveforms, and the average value was used for analysis. An UA hPSV-DT value below the 5th percentile for gestational age, according to the nomogram by Bustos JC et al., was considered abnormal. Standard UA Doppler indices, including pulsatility index (PI) and systolic/diastolic ratio (S/D), were also recorded, along with uterine artery (UtA) and middle cerebral artery (MCA) Doppler parameters.

Maternal demographic characteristics, GDM management type, OGTT results, and HbA1c levels were recorded. Fetal biometry, estimated fetal weight (EFW), and abdominal circumference (AC) percentiles were calculated. Neonatal outcomes were prospectively collected, including birth weight, gestational age at delivery, Apgar scores, and the presence of composite adverse perinatal outcome (CAPO), defined as one or more of the following: fetal distress, neonatal intensive care unit (NICU) admission, respiratory distress syndrome (RDS), transient tachypnea of the newborn (TTN), need for continuous positive airway pressure (CPAP) or mechanical ventilation, neonatal sepsis, phototherapy requirement, or neonatal hypoglycemia. As the main aim of this study was to evaluate the predictive role of UA hPSV-DT in GDM pregnancies, subgroup analyses were restricted to women with GDM. All non-diabetic controls had UA hPSV-DT values within the normal range for gestational age and were therefore not included in the abnormal vs. normal subgroup comparisons.

This study was conducted in accordance with the Declaration of Helsinki and approved by the Ankara Etlik City Hospital Ethics Committee (approval number: AESH-BADEK–2025-0048). Written informed consent was obtained from all participants before enrollment.

### Statistical Analysis

Statistical analysis was performed using IBM SPSS Statistics version 30.0 (IBM Corporation, Armonk, NY, USA). The Kolmogorov–Smirnov test was used to assess the normality of data distribution. Descriptive statistics of continuous variables are presented as mean ± standard deviation for normally distributed data, and as median (interquartile range) for non-normally distributed data. Comparisons of continuous variables were conducted using Student’s *t*-test for normally distributed variables and the Mann–Whitney U test for non-normally distributed variables. Categorical variables are presented as numbers and percentages (*n*, %) and were compared using Pearson’s Chi-square test or Fisher’s Exact test where appropriate. This approach aimed to minimize overfitting while addressing potential confounding. ROC curve analysis was used to calculate and compare the AUC and to identify optimal cut-off values using the Youden index. A *p*-value of <0.05 was considered statistically significant.

## 3. Results

A total of 120 singleton pregnancies were analyzed, including 30 with insulin-regulated gestational diabetes mellitus (IRGDM), 30 with diet-regulated gestational diabetes mellitus (DRGDM), and 60 healthy controls. Maternal age, body mass index, gravidity, parity, and gestational age at examination were similar among the three groups (*p* > 0.05). Conventional Doppler indices of the umbilical artery (UA), uterine artery (UtA), and middle cerebral artery (MCA) did not differ significantly between groups. However, UA hPSV-DT was significantly shorter in both GDM subgroups compared to controls, with the lowest median value observed in the IRGDM group (171 ms vs. 184 ms vs. 227 ms, *p* = 0.006) (Table 1).

All non-diabetic control participants had UA hPSV-DT values within the normal range; therefore, subgroup analyses were limited to GDM patients. When participants were classified according to UA hPSV-DT status, 26 women (43.3%) had abnormal values (<5th percentile). Compared to the normal UA hPSV-DT group, these women had higher estimated fetal weight and abdominal circumference percentiles, greater amniotic fluid depth, higher 1 h and 2 h OGTT glucose levels, and elevated HbA1c (all *p* < 0.01). The need for insulin therapy was also more frequent in the abnormal UA hPSV-DT group (69.2% vs. 32.4%, *p* = 0.010) (Table 2).

Adverse perinatal outcomes were more common in the abnormal UA hPSV-DT group. These pregnancies had earlier delivery (37 vs. 38 weeks, *p* = 0.005) and a higher incidence of composite adverse perinatal outcome (CAPO) (73.1% vs. 11.8%, *p* < 0.001). NICU admission (61.5% vs. 14.7%, *p* < 0.001), transient tachypnea of the newborn (30.7% vs. 8.8%, *p* = 0.021), need for continuous positive airway pressure (34.6% vs. 8.8%, *p* = 0.013), phototherapy (37.5% vs. 5.9%, *p* = 0.005), and neonatal hypoglycemia (33.3% vs. 5.9%, *p* = 0.011) were significantly more frequent in the abnormal group (Table 3).

**Figure 2:**
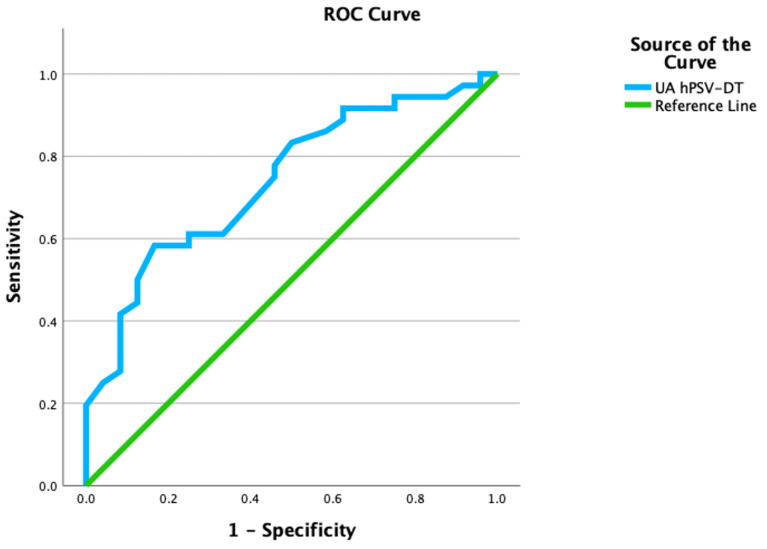
ROC Curve Demonstrating the Predictive Accuracy of hPSV-DT in Identifying Patients at Risk for CAPO. ROC curve demonstrating the predictive accuracy of umbilical artery half peak systolic velocity deceleration time (UA hPSV-DT) for identifying pregnancies with gestational diabetes mellitus at risk for composite adverse perinatal outcome (CAPO). The optimal cut-off value of <181 ms yielded an area under the curve (AUC) of 0.741, with 70.3% sensitivity and 66.7% specificity.

Receiver operating characteristic (ROC) curve analysis identified a UA hPSV-DT cut-off value of <181 ms as predictive of CAPO, with 70.3% sensitivity, 66.7% specificity, and an area under the curve (AUC) of 0.741 (95% CI 0.615–0.866, *p* < 0.001) (Table 4, Figure 2).

In multivariable logistic regression analysis including UA hPSV-DT, HbA1c, and insulin therapy, abnormal UA hPSV-DT remained an independent predictor of CAPO (aOR 3.01, 95% CI 1.21–5.01, *p* = 0.002). Higher HbA1c levels were also significantly associated with an increased risk of CAPO (aOR 2.34, 95% CI 1.11–3.99, *p* = 0.031). Insulin therapy showed a borderline but significant association (aOR 2.53, 95% CI 0.98–3.78, *p* = 0.043). (Table 5)

## 4. Discussion

To our knowledge, this is the first study to investigate the predictive value of UA hPSV-DT in pregnancies complicated by GDM. Median UA hPSV-DT values were 171 ms in IRGDM, 184 ms in DRGDM, and 227 ms in healthy controls (*p* = 0.006). An abnormal UA hPSV-DT (<5th percentile for gestational age) was present in 43.3% of GDM cases and was associated with higher estimated fetal weight and abdominal circumference percentiles, increased amniotic fluid, elevated 1 h and 2 h OGTT glucose levels, higher HbA1c, and more frequent insulin therapy. Pregnancies with abnormal UA hPSV-DT had a 73.1% incidence of CAPO compared to 11.8% in those with normal values (*p* < 0.001). ROC analysis identified a cut-off of <181 ms for predicting CAPO, with a sensitivity of 70.3%, a specificity of 66.7%, and an AUC of 0.741. In multivariate analysis, abnormal UA hPSV-DT remained an independent predictor of CAPO (adjusted OR 3.51, 95% CI 1.41–5.51, *p* = 0.004) alongside elevated HbA1c and insulin requirement.

GDM induces a cascade of metabolic and vascular alterations that adversely affect placental structure and function [12]. Maternal hyperglycemia drives fetal hyperinsulinemia and accelerated growth but paradoxically creates a chronic intrauterine imbalance between oxygen supply and demand. Elevated fetal metabolic rate increases oxygen consumption, which can push the placenta into relative hypoxia despite adequate or increased size [13,14]. These metabolic stresses are compounded by placental oxidative stress, inflammation, and endothelial dysfunction, often accompanied by incomplete spiral artery remodeling and increased uteroplacental resistance [15]. Histopathological studies confirm that GDM placentas, though frequently enlarged, commonly exhibit villous immaturity, microvascular lesions, fibrin deposition, edema, calcifications, and vascular congestion—changes that impair vascular compliance and slow intervillous blood flow [16]. In severe cases, intermittent flow disturbances or even transient flow cessation can occur, leading to fetal distress or growth restriction despite normal or large fetal size [17].

UA hPSV-DT is directly influenced by placental vascular resistance and compliance, measuring the time for systolic blood flow to fall to half its peak velocity [9]. A longer deceleration time reflects sustained diastolic flow in a compliant, low-resistance placental bed, whereas a shorter time indicates rapid post-systolic flow decline due to higher downstream resistance. In GDM pregnancies with poor outcomes, maternal endothelial dysfunction and diabetic vasculopathy likely stiffen the placental vessels, causing systolic flow to dissipate more quickly and producing abnormally short hPSV-DT values even when UA-PI remains normal [18,19]. Experimental and clinical data show that diabetic placentas can have reduced perfusion and a tendency toward increased UA resistance despite subtle or absent changes in conventional Doppler indices [17,20]. Chronic fetal hypoxemia in poorly controlled GDM may also trigger brain-sparing redistribution, further increasing UA resistance and diminishing diastolic flow—physiological changes sensitively captured by hPSV-DT [18,19]. Thus, our observation of shortened hPSV-DT in GDM pregnancies with adverse outcomes is biologically plausible and consistent with underlying subclinical uteroplacental underperfusion, mirroring pathophysiological patterns seen in high-resistance states such as preeclampsia and fetal growth restriction.

UA hPSV-DT is a relatively novel Doppler metric, and our study is the first to evaluate its predictive value specifically in GDM pregnancies. Nevertheless, a number of prior studies in other contexts have laid the groundwork for understanding this parameter’s significance. Bustos et al. first described the hPSV-DT in a cohort of over 500 normal pregnancies, demonstrating that it increases linearly with advancing gestational age (approximately 6–7 milliseconds per week). They reported that measuring hPSV-DT is highly reproducible and, importantly, independent of fetal heart rate. This independence from heart rate was highlighted by their observation that in fetuses with extreme bradycardia (such as complete heart block), hPSV-DT remained within normal range (above the 5th percentile for gestational age) even though conventional impedance indices (like UA-PI) were artifactually elevated by the prolonged cardiac cycle. In other words, hPSV-DT can more purely reflect placental function without being confounded by fetal heart rate variations, which is a key advantage over traditional Doppler ratios [9].

Following its introduction, hPSV-DT has been investigated most extensively in settings of placental insufficiency. In fetal growth restriction (FGR), where increased placental vascular resistance is the central pathology, hPSV-DT has shown remarkable diagnostic and prognostic utility. Bustos et al. studied 266 high-risk pregnancies with FGR and found that 87% of moderately growth-restricted and 98% of severely FGR fetuses had UA hPSV-DT values below the 5th percentile for gestation. Shortened deceleration time correlated strongly with adverse outcomes: 94% of fetuses with an hPSV-DT < 90 ms had a poor perinatal outcome, such as perinatal death or prolonged NICU admission. None of the FGR fetuses in that series had an hPSV-DT < 70 ms, suggesting that such an extremely short deceleration time is rarely seen except in agonal states. Indeed, the authors proposed absolute thresholds for risk stratification in FGR: hPSV-DT < 90 ms was associated with imminent risk of intrauterine demise, and < 70 ms was deemed likely “incompatible with intrauterine life”. They further noted that even among the sickest FGR fetuses (those with absent or reversed end-diastolic flow in the UA), the perinatal mortality rate differed significantly by hPSV-DT—51% when hPSV-DT was ≤90 ms versus 23% when >90 ms. These findings firmly establish hPSV-DT as an indicator of placental failure severity; the shorter the deceleration time, the worse the placental perfusion and the higher the risk of fetal demise [10].

In preeclampsia, a disorder characterized by placental hypoperfusion, recent studies also support the value of UA hPSV-DT. Tokalıoğlu et al. reported that preeclamptic pregnancies with an abnormally low hPSV-DT (<5th percentile for gestational age) had significantly poorer neonatal outcomes compared to those with normal values, including lower birth weights, earlier gestational age at delivery, lower Apgar scores, and more acidotic cord pH measurements. Adverse outcomes such as NICU admission, RDS, <34-week delivery, and low birth weight were 2–3 times more common in the abnormal group. Importantly, conventional UA-PI > 95th percentile detected only 2 of 23 newborns requiring NICU care, whereas hPSV-DT < 5th percentile identified 16 of 23 (69%). Diagnostic performance analysis found an optimal cut-off of ~221.5 ms (≈5th percentile at 32–34 weeks), with 82.6% sensitivity and 69.1% specificity for predicting NICU admission [21]. This higher absolute threshold compared to severe early-onset FGR (e.g., ~90 ms) illustrates that hPSV-DT cut-offs are context dependent. In chronic, extreme placental insufficiency, hPSV-DT can fall below 100 ms, while in conditions like late-onset preeclampsia or GDM, placental resistance is often only moderately elevated; thus, even a milder shortening from gestational norms can still predict adverse outcomes. Our GDM findings fit this pattern: abnormal hPSV-DT values corresponded to moderate deceleration time shortening (in the range of a few hundred milliseconds rather than <100 ms), indicating early placental dysfunction with significant clinical consequences.

Beyond FGR and hypertensive disorders, aneuploidy also provides an important comparative context for UA hPSV-DT. Fetuses with trisomy 21 frequently exhibit abnormal UA Doppler findings even in the absence of overt preeclampsia or severe growth restriction. Bustos et al. evaluated UA-PI and hPSV-DT in second- and third-trimester fetuses with trisomy 21 and demonstrated that as gestation advanced, an increasing proportion developed abnormally short hPSV-DT values, despite only about 29% being born small for gestational age (SGA). By the late third trimester, 91% of fetuses with trisomy 21 had hPSV-DT values below the normal range, and 78% had elevated UA-PI. Mean UA hPSV-DT z-scores were approximately –2.18, equivalent to two standard deviations below normal at the final assessment. Notably, virtually all Down syndrome fetuses born SGA had abnormal hPSV-DT (100%), but Doppler abnormalities were also present in many appropriately grown or even large fetuses. The authors suggested that placental vascular resistance increases progressively in trisomy 21, beginning in the mid-second trimester and becoming more pronounced by term. However, unlike classical FGR due to severe placental insufficiency, the impact on fetal growth was modest, with mean birthweights only slightly below average. This supports the interpretation that trisomy 21 placentas can be functionally impaired with increased resistance without proportional growth failure, possibly due to compensatory factors such as reduced fetal metabolic demand or alternative placental developmental pathways [22]. Importantly, the ability of hPSV-DT to reveal these hemodynamic alterations in the absence of growth restriction highlights its sensitivity as a Doppler marker. Similarly, studies of trisomy 18 and 13 have documented markedly elevated placental resistance and very short hPSV-DT, reflecting their frequent association with severe early-onset FGR and placental failure [23]. Collectively, evidence from aneuploidies, together with findings in normal pregnancies, FGR, and preeclampsia, supports a consistent pattern: UA hPSV-DT is a robust indicator of fetoplacental circulation efficiency. When the placenta is healthy and low-resistance, hPSV-DT lengthens predictably with gestation, whereas in pathologically high-resistance states it shortens, often preceding or outperforming traditional Doppler indices in identifying fetuses at risk.

Prior to our study, hPSV-DT had not been specifically investigated in diabetic pregnancies. Meta-analyses of Doppler findings in GDM reported no consistent differences in UA-PI compared to non-diabetic controls, although slight increases in UA-RI and uterine artery PI have been observed. This supports the notion that well-controlled GDM often maintains normal placental resistance [8]. Our data refine this understanding: within the GDM cohort, those with abnormally short hPSV-DT represented a high-resistance subgroup with a significantly increased risk of adverse perinatal outcomes. Unlike the extreme shortening seen in severe FGR (<90 ms), risk in GDM was linked to more moderate shortening—defined relative to gestational percentiles (e.g., <5th percentile, ~<180–200 ms depending on gestational age). This suggests that in GDM, even modest deviations from normal hPSV-DT values can have important clinical implications.

Our findings suggest that incorporating UA hPSV-DT measurement into routine Doppler assessment for GDM pregnancies could enable earlier detection of incipient placental dysfunction, even in well-grown fetuses without overt preeclampsia. In such cases, an abnormal hPSV-DT might prompt more frequent monitoring or discussions on earlier delivery to prevent fetal distress or stillbirth, similar to its proposed role in preeclampsia for guiding delivery timing [21]. This approach could help tailor management for heterogeneous GDM cases—identifying high-risk patients (e.g., insulin-dependent, hypertensive, obese) before clinical signs appear, while reassuring continued observation in those with normal or improving values. Given GDM’s association with stillbirth, especially in poorly controlled late pregnancy, hPSV-DT may help distinguish pregnancies with true placental oxygen delivery compromise from those whose risk is primarily macrosomia or birth trauma, influencing decisions on antenatal corticosteroids or delivery mode. The clinical value is supported by the study’s strengths: to our knowledge, this is the first prospective investigation of hPSV-DT in GDM; all Doppler measurements were performed using a standardized protocol by a single experienced perinatologist, ensuring consistency; a broad dataset encompassing Doppler, maternal metabolic parameters, and detailed neonatal outcomes was analyzed; and independent associations were confirmed through multivariate logistic regression. The practicality of hPSV-DT—being numeric, objective, reproducible, and obtainable from the same UA waveform used for PI—means it could be seamlessly integrated into third-trimester risk stratification, enabling proactive, evidence-based interventions that may reduce perinatal morbidity and mortality in diabetic pregnancies.

This study has several limitations that should be considered when interpreting the results. First, the relatively small sample size and single-center design may limit the generalizability of the findings, as patient demographics and management protocols may differ in other settings. In particular, the small number of CAPO events restricted our ability to fully adjust for confounding variables. To minimize overfitting, we therefore used a parsimonious multivariable logistic regression model including only UA hPSV-DT, HbA1c, and insulin therapy, which we considered the most clinically relevant covariates. Second, although all UA hPSV-DT measurements were performed by a single experienced perinatologist using a standardized protocol, Doppler assessment remains operator-dependent, and inter-observer reproducibility was not formally evaluated. Third, measurements were obtained at a single time point in the third trimester, so longitudinal changes in hPSV-DT in response to glycemic control or disease progression could not be assessed. Fourth, the composite adverse perinatal outcome included events of varying clinical severity, which may dilute associations with the most critical outcomes. Another limitation is that UA hPSV-DT was not directly compared with standard antenatal surveillance methods such as amniotic fluid volume assessment, biophysical profile, or serial Doppler testing. Finally, as an observational study, our findings establish an association but cannot confirm causality between shortened hPSV-DT and adverse perinatal outcomes in GDM. Future studies should aim to validate these findings in larger, multicenter cohorts and across diverse populations. Longitudinal designs could help determine whether changes in hPSV-DT over time predict worsening placental function or adverse outcomes. Research correlating hPSV-DT with placental histopathology and fetal biomarkers may clarify the underlying mechanisms, while interventional trials could explore whether intensified surveillance or earlier delivery in cases with abnormal hPSV-DT improves perinatal outcomes.

## 5. Conclusions

In conclusion, this study adds to a growing body of evidence that UA hPSV-DT is a valuable Doppler index for fetoplacental well-being. Our results in gestational diabetes mellitus expand the relevance of hPSV-DT beyond traditionally studied conditions, highlighting its promise as an early placental “stress test.” With further research to validate and refine its use, hPSV-DT has the potential to improve risk assessment and guide interventions in GDM pregnancies, ultimately aiming to enhance perinatal outcomes for this at-risk population.

## Figures and Tables

**Table 1:** Comparison of Demographic and Doppler Ultrasonography Parameters Between IRGDM, DRGDM, and Control Groups.

	IRGDM n = 30 (25%)	DRGDM n = 30 (25%)	Control n = 60 (50%)	*p* Value
Maternal age (year)	32.2 ± 5.1	33.1 ± 5.9	31.5 ± 5.4	0.183
BMI (kg/m^2)^	29.5 ± 5.2	28.3 ± 6.9	28.7 ± 5	0.156
Gravida (n)	2 (2)	2 (2)	1 (2)	0.326
Parity (n)	1 (2)	0 (2)	0 (2)	0.270
Gestational age at examination	34 (4)	33 (4)	34 (3)	0.437
UA S/D	2.33 (0.76)	2.34 (0.54)	2.46 (0.53)	0.248
UA PI	0.85 (0.31)	0.81 (0.21)	0.89 (0.25)	0.321
UtA S/D	1.94 (0.59)	1.98 (0.45)	1.84 (0.56)	0.228
UtA PI	0.75 (0.30)	0.73 (0.35)	0.83 (0.50)	0.577
MCA PSV	43.13 (15.08)	49.39 (11.67)	47.80 (16.29	0.431
MCA S/D	4.38 (2.19)	4.55 (1.34)	4.96 (2.33)	0.295
MCA PI	1.55 (0.41)	1.61 (0.31)	1.60 (0.35)	0.408
UA hPSV-DT(ms)	171 (46)	184 (64)	227 (59)	0.006 ^a,b,c^

Data are presented as mean ± standard deviation or median (interquartile range), as appropriate. Comparisons among the three groups (IRGDM: Insulin-Regulated Gestational Diabetes Mellitus, DRGDM: Diet-Regulated Gestational Diabetes Mellitus, and Control) were conducted using one-way ANOVA or Kruskal–Wallis test, depending on data distribution. ^a^:The difference between Group IRGDM and Group DRGDM is significant, ^b^: The difference between Group DRGDM and Group Control is significant, ^c^: The difference between Group IRGDM and Group Control is significant UA: Umbilical artery; UtA: Uterine artery; MCA: Middle cerebral artery; BMI: Body mass index; PSV: Peak systolic velocity; PI: Pulsatility index; S/D: Systolic/diastolic ratio.

**Table 2:** Comparison of Maternal Metabolic, Fetal Biometric, and Doppler Parameters Between Groups with Normal and Abnormal Umbilical Artery Half Peak Systolic Velocity Deceleration Time (hPSV-DT).

	Abnormal hPSV-DT n:26 (43.3%)	Normal hPSV-DT n:34 (56.7%)	*p* Value
Gestational age at diagnosis (week)	32 (4)	34 (2)	0.329
EFW percentile	85 (11)	68 (22)	0.004
AC percentile	90 (15)	73 (27)	0.010
DeepVertical Pocket (mm)	100 (31)	66 (35)	0.003
Peak systolic velocity (cm/s)	45.5 (11.8)	48.4 (11.3)	0.066
UA hPSV-DT (ms)	165 (39)	203 (63)	<0.001
OGTT 75 g– Fasting (mg/dL)	99 (16)	92 (14)	0.057
OGTT 75 g– 1 h (mg/dL)	218 (21)	189 (28)	0.001
OGTT 75 g– 2 h (mg/dL)	134 (34)	117 (25)	0.008
Hba1c (%)	6.8 (0.9)	5.6 (0.4)	<0.001
Insulin therapy, n (%)	69.2%	32.4%	0.010

Data are presented as mean ± standard deviation or median (interquartile range), as appropriate. Comparisons between groups with normal and abnormal umbilical artery half peak systolic velocity time (UAHPSVT) were performed using Student’s *t*-test or Mann–Whitney *U* test, depending on data distribution. UAHPSVT: Umbilical artery half peak systolic velocity time; EFW: Estimated fetal weight; AC: Abdominal circumference; OGTT: Oral glucose tolerance test; HbA1c: Hemoglobin A1c.

**Table 3:** Comparison of Maternal and Neonatal Outcomes Between Pregnancies With Normal and Abnormal Umbilical Artery Half Peak Systolic Velocity Deceleration Time (hPSV-DT).

	Abnormal hPSV-DT n:26 (43.3%)	Normal hPSV-DT n:34 (56.7%)	*p* Value
Gestational age at delivery (week)	37 (3)	38 (2)	0.005
Prematurity (<37 weeks)	8 (30.8%)	4 (11.8%)	0.104
Birth weight (g)	3600 (713)	3335 (483)	0.191
Fetal distress	7 (26.9%)	5 (14.7%)	0.397
Cesarean section	16 (61.5%)	20 (58.8%)	0.004
Apgar score at 1st minute	8 (2)	8 (1)	0.117
Apgar score at 5th minute	9 (2)	9 (0)	0.111
CAPO	19 (73.1%)	4 (11.8%)	<0.001
NICU admission	16 (61.5%)	5 (14.7)	<0.001
Transient tachypnea of the newborn	8 (30.7%)	3 (8.8)	0.021
Neonatal sepsis	1 (3.8%)	1 (2.9%)	0.846
Respiratory distress syndrome	6 (23.0%)	4 (11.8%)	0.091
Continues positive airway pressure	9 (34.6%)	3 (8.8)	0.013
Mechanical ventilation	5 (20.8%)	4 (11.8%)	0.467
Phototherapy for neonates	9 (37.5%)	2 (5.9%)	0.005
Neonatal hypoglycemia	8 (33.3%)	2 (5.9%)	0.011
Surfactant Use	1 (4.2%)	0 (0.0)	0.414

Data are presented as mean ± standard deviation or median (interquartile range), and frequencies are given as n (%). *p* values were calculated using Student’s *t*-test, Mann–Whitney *U* test, or chi-square test, as appropriate. NICU: Neonatal intensive care unit; CAPO: Composite adverse perinatal outcome.

**Table 4:** Predictive Performance of Half Peak Systolic Velocity Deceleration Time (hPSV-DT) for CAPO Assessed by ROC Analysis.

	Cut-off *	Sensitivity	Specificity	AUC	%95 CI	*p*-Value
*UA* hPSV-DT	<181	70.3%	66.7%	0.741	0.615–0.866	<0.001

* Cut-off values were found according to Youden index. AUC: Area under the curve, CI: Confidence Interval, hPSV-DT: Half Peak Systolic Velocity Deceleration Time.

**Table 5:** Multivariate Logistic Regression Analysis of Factors Associated with CAPO.

Variable	aOR (95% CI)	*p* Value
UA hPSV-DT	3.01 (1.21–5.01)	**0.004**
Hba1c	2.34 (1.11–3.99)	**0.031**
Insulin therapy	2.53 (0.98–3.78)	**0.043**

All variables listed were included in the regression model. Statistically significant results are shown in bold (*p* < 0.05).

## Data Availability

If requested, data can be shared by the corresponding author with patient names and IDs anonymized.

## Abbreviations

The following abbreviations are used in this manuscript:

AC	Abdominal Circumference
AUC	Area Under the Curve
CAPO	Composite Adverse Perinatal Outcome
CI	Confidence Interval
CPAP	Continuous Positive Airway Pressure
DRGDM	Diet-Regulated Gestational Diabetes Mellitus
EFW	Estimated Fetal Weight
FGR	Fetal Growth Restriction
GDM	Gestational Diabetes Mellitus
hPSV-DT	Half-Peak Systolic Velocity Deceleration Time
IADPSG	International Association of the Diabetes and Pregnancy Study Groups
IRGDM	Insulin-Regulated Gestational Diabetes Mellitus
MCA	Middle Cerebral Artery
NICU	Neonatal Intensive Care Unit
OGTT	Oral Glucose Tolerance Test
OR	Odds Ratio
PI	Pulsatility Index
RDS	Respiratory Distress Syndrome
ROC	Receiver Operating Characteristic
SD	Standard Deviation
S/D ratio	Systolic/Diastolic Ratio
TTN	Transient Tachypnea of the Newborn
UA	Umbilical Artery
UtA	Uterine Artery
